# High-Resolution Infrared Reflectance Distribution Measurement Under Variable Temperature Conditions

**DOI:** 10.3390/s24216780

**Published:** 2024-10-22

**Authors:** Yujian Yang, Yao Li, Ang Huang, Fanshan Meng, Jinghui Wang, Wei Dong, Yiwen Li

**Affiliations:** 1 College of Mechanical and Power Engineering, Shenyang University of Chemical Technology, Shenyang 110142, China; zhaogefengqi@163.com; 2 Science and Technology on Plasma Dynamic Laboratory, Airforce Engineering University, Xi’an 710038, China; lee_yiwen@163.com; 3 Division of Thermophysics Metrology, National Institute of Metrology, Beijing 100029, China; huangang@nim.ac.cn (A.H.); mfs18722117725@163.com (F.M.); wangjh@nim.ac.cn (J.W.); dongw@nim.ac.cn (W.D.)

**Keywords:** infrared BRDF, variable temperature, robotic arm

## Abstract

The bidirectional reflectance distribution function (BRDF) can effectively characterize the reflectance properties of a target, which can be used to correct infrared remote sensing data and improve the accuracy of remote sensing measurements. When the surface temperature changes, the reflectance characteristics of the target usually change, and it is necessary to carry out BRDF measurements under variable temperature conditions. In this paper, a variable-temperature infrared BRDF measurement system based on a robotic arm is developed to realize high-resolution wide-temperature region measurement of BRDF. To improve the measurement accuracy, the shaping optical path was used to expand the laser beam, combined with the laser level to accurately adjust the three-dimensional coordinates of the robotic arm, and the dichotomy method is used to calibrate the detector nonlinearly. A portable heater suitable for the mechanical arm corner mechanism is developed, and fast and high-precision temperature control is realized by proportional integral derivative (PID) control. The specular and diffuse BRDF distributions were measured at room temperature to verify the effectiveness of the system. The BRDF distribution of SUS314 stainless steel samples with different roughness is measured during two temperature increases from 20 °C to 1000 °C, and the changing rule of BRDF under variable temperature environment is summarized, which provides technical support for evaluating the optical properties of high-temperature materials and improving the measurement accuracy of remote sensing data.

## 1. Introduction

The bidirectional reflectance distribution function (BRDF) is a basic physical parameter used to represent the reflectance characteristics of a material surface and is defined as the ratio of the reflected luminance to the incident illuminance in each direction [1]. In the field of environmental monitoring, atmospheric or terrain-related data are obtained through remote sensing satellites, and coupled atmosphere–terrain–BRDF correction products have been developed to realize high-precision monitoring of changes in surface coverage and airflow status [2,3,4,5,6,7,8]. However, these studies mainly focus on the measurement of atmospheric temperature conditions; when there are special conditions such as burning and freezing, the temperature changes dramatically, and the surface BRDF changes, which may cause the target reflectance characteristics analysis to have a large deviation [9,10].

BRDF measurement systems have different types of rotation mechanisms, including unmanned aerial vehicle (UAV), closed-field, multi-rotor, and robotic arm, which are mainly measured under ambient temperature. BRDF systems based on UAV and closed-field are used to detect scattered light at different reflection angles by changing the detector at different positions in the hemispherical space of the target sample while the sample and light source are fixed. The UAV can stably hover at any position in the BRDF hemispherical space to realize BRDF measurements of different reflection angles [11,12,13,14,15]. The systems are mainly used for vegetation monitoring and image correction and natural light conditions [16,17,18]. Zhao et al. [19] used BRDF to detect the scattered light at the Dunhuang Radiation Correction Field in China. The reflected zenith angle ranged from 0° to 36° with an angular resolution of 6°; the reflected azimuth angle ranged from 0° to 330° with an angular resolution of 30°. However, the spatial resolution was low and the BRDF measurement could not cover the whole hemispheric space. In a closed-field-based BRDF measurement system, the incident light source is kept relatively stationary with the sample, and the detector detects the scattered light with different reflection angles from a hemispherical or global surface field [20,21,22]. Zou et al. developed a hemispherical closed-field BRDF measurement system [23]. The wavelength range of the light source was 380~780 nm, which enables fast BRDF measurements of anisotropic samples in the range of 0°~80° zenith angle and 0°~360° azimuthal angle, with an angular resolution of 1°. Closed-field structures are expensive, and their parameters are difficult to modify.

BRDF rotation strategy of multi-rotor and robotic arms causes different reflection angles through the relative motion between the light source, detector, and sample. The University of Aalto has developed a BRDF measurement device with a multi-axis rotating angular mechanism with one degree of freedom for the light source and two degrees of freedom for the detector. The detection ranges were −180°~+180° for reflected azimuth and −90°~+90° for reflected zenith, and the spatial angular resolution was 0.09° [24]. The parameters of the multi-rotor can be easily changed in measurements, but it is complicated to operate and prone to introduce mechanical errors. Mechanical arm cornering mechanisms through mechatronics can be used to achieve synchronous control of multiple degrees of freedom; they are easy to operate and have higher motion accuracy [25,26,27,28,29,30,31,32,33]. For example, Rabal et al. [34,35] developed a BRDF measurement system based on a six-axis robotic arm turning mechanism, a reflection zenith angle range of −70°~+70°, and a reflection azimuth angle range of 0°~360°. A classification of BRDF measurement devices based on different types of cornering mechanisms under ambient conditions is described, and the parameters of each device are summarized in Table 1.

High-temperature BRDF measurements are mainly carried out by laser heating and furnace heating [36,37,38,39]. Bailey et al. [36] heated stainless steel and copper samples by laser heating, and the heating and incident light source was a Nd:YAG solid-state laser with a power and wavelength of 230 W and 1.06 μm, respectively, which heated the samples up to 850 °C. The scattered light was detected by imaging the scattered light from the samples onto a Lambertian screen, and the dynamic change of the scattered light on the Lambertian screen was measured in real time by an infrared camera. The BRDF values of the samples, which dynamically changed with increasing temperature, were calculated. Researchers at Johns Hopkins University used high-energy laser irradiation on the back of the sample for heating; another laser beam irradiated the measured surface of the stainless-steel sample by incident light heating. The heating light source was a 50 W CO_2_ gas laser, and the heating temperature could reach 500 °C [39].

In BRDF measurement devices based on furnace heating, the furnace is usually designed for multi-axis cornering mechanisms, where the sample is heated and stabilized at a fixed temperature compared to the continuous heating of a high-energy laser. Zhao et al. [40,41] designed a cylindrical heating furnace for the BRDF measurement device of a three-degree-of-freedom multi-rotor angular mechanism. The heating furnace can heat the sample from room temperature to 500 °C. A self-tuning PID controller is used for temperature control, with a control accuracy of ±1 °C. The heating furnace is designed for the measurement of the BRDF. The incident light source wavelength in the measurement system is 0.6328~10.6 μm. Chen et al. [42] designed a flat plate heater with a maximum heating temperature of 800 °C for the BRDF measurement device of a multi-rotor angular mechanism, which can realize the BRDF measurement within the range of incidence and reflection angles up to *θ_imax_* = *θ_rmax_* = 78° due to the flat plate heater’s thermal insulation plane being higher than the measured surface of the sample. Liu et al. [43] established a model of alumina reflection characteristics by a similar variable-temperature BRDF measurement device with polarized BRDF measurements in the range of 25~800 °C on alumina with different roughness. The heating methods, maximum heating temperatures, and applicable corner mechanisms of the variable temperature BRDF measuring devices are summarized in Table 2.

We chose a mechanical arm corner mechanism and used a portable heater specially designed for the mechanical arm corner mechanism to develop a variable-temperature infrared BRDF measurement system that realizes a high resolution from room temperature to 1000 °C. The effectiveness of the system is verified by measuring the BRDF of specular and diffuse reflective materials at room temperature. Under variable temperature conditions, the mechanism of the temperature effect on BRDF is investigated by measuring the change in hemispherical space BRDF of stainless steel samples with different roughness during the initial and secondary heating processes, and by comparing the surface microstructure of the samples at ambient temperature or after initial heating and secondary heating.

## 2. System Development

### 2.1. Measuring Principle

Existing BRDF measurement methods are categorized into relative and absolute measurement methods. The relative measurement method has lower requirements for the experimental measurement system, but this method must rely on a standard reference plate, which is not suitable for all wavelengths or high-temperature conditions, and is not applicable to infrared wavelengths, variable-temperature conditions, and hemispherical space completion measurement on the sample surface [44]. The absolute measurement method is based on the BRDF definition, with no reference standard. The measurement system developed in this paper is based on the absolute measurement method.

BRDF is a physical parameter characterizing the spatial reflection properties of a target surface, which is defined as the ratio of the reflected irradiance d*L_r_*[W·m^−2^·sr^−1^·nm^−1^] in a certain reflection direction (*θ_r_*, *ϕ_r_*) to the irradiance d*E_i_*[W·m^−2^·nm^−1^] in the incidence direction (*θ_i_, ϕ_i_*), *f*_d_(sr^−1^). The expression for BRDF is given by:

(1) $$f_{d}\left(\theta_{i},\phi_{i},\theta_{r},\phi_{r},\lambda \right)=\frac{dL_{r}\left(\theta_{i},\phi_{i},\theta_{r},\phi_{r},\lambda \right)}{dE_{i}\left(\theta_{i},\theta_{r},\lambda \right)}$$

where *i* and *r* denote incident and reflected, respectively, *θ_i_* and *θ_r_* denote incident and reflected zenith angles, respectively, *ϕ_i_* and *ϕ_r_* denote incident and reflected azimuth angles, respectively, and *λ* denotes wavelength.

The definition in Equation (1) is in differential form and cannot be measured directly. BRDF measurement is usually in the form of power probing. In this paper, the BRDF is measured at different slice temperatures *T*. The BRDF expression after introducing the temperature variable is:

(2) $$f_{d}\left(\theta_{i},\varphi_{i},\theta_{r},\varphi_{r},\lambda ,T\right)=\frac{P_{r}\left(\theta_{i},\phi_{i},\theta_{r},\phi_{r},\lambda ,T\right)}{P_{i}\left(\theta_{i},\theta_{r},\lambda \right)\left(\Omega_{r}\cdot \cos\theta_{r}\right)}$$

where *T* is the sample surface temperature, *P_r_* is the reflected power in the reflection direction (*θ_r_*, *ϕ_r_*), *P_i_* is the incident power in the incidence direction (*θ_i_*, *ϕ_i_*), and $\Omega_{r}$ is the steradian angle of the detector aperture, denoted as:

(3) $$\Omega_{r}=A/D^{2}$$

where *A* is the detection area and *D* is the distance from the sample center to the detector.

### 2.2. Global Design

**Figure 1:**
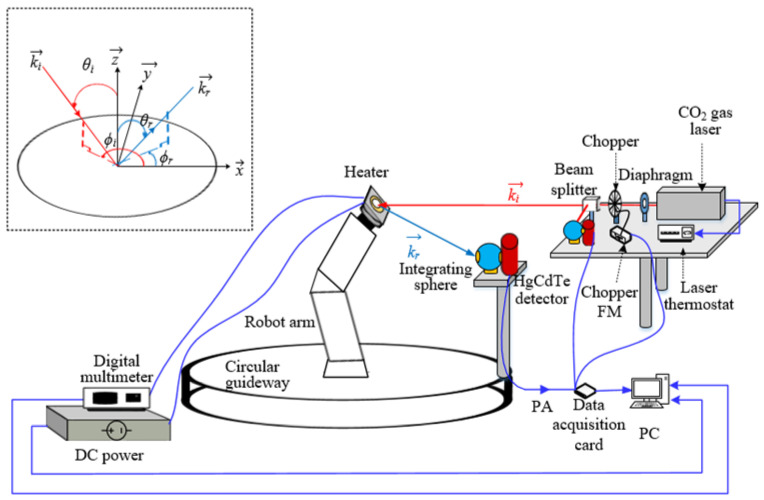
Schematic of infrared BRDF measurement system with variable temperature.

A BRDF measurement system based on a robotic arm was developed according to Equation (2). The system included a laser source module, a motion module, a signal detection and processing module, and a temperature controlling module, as shown in Figure 1. The laser source module provides an infrared light source, the motion module realizes the attitude change of the sample with different angles of incidence and reflection, the temperature controlling module realizes the heating and temperature control of the sample, and the signal detection and processing module realizes the detection of the incident light and the scattered light and is used for data storage and post-processing. The model and main parameters of all equipment in the system are shown in Table 3.

The laser light source module employs a mid-infrared CO_2_ gas laser with a wavelength of 10.6 μm [45,46]. The motion module comprises a six-axis robotic arm and a circular guideway, with the heater and the sample secured in the gripper of the robotic arm. The HgCdTe detector is fixed in the circular guideway, enabling the continuous collection of scattered light at varying reflection angles through the uniform rotation of the circular guideway. The signal detection and processing module mainly contains the detector, data acquisition card, and calculation program; the solid angle of the detector is 0.0013 sr, the response wavelength range is 2~13 μm, and the sampling rate of the acquisition card is 102.4 kHz. The sampled data were transmitted to the host PC for BRDF real-time calculation. The sample can be heated to up to 1000 °C by the temperature-controlling module, with an error of ±0.7 °C.

### 2.3. Laser Source Module

#### 2.3.1. Laser Optical Path Design

**Figure 2:**
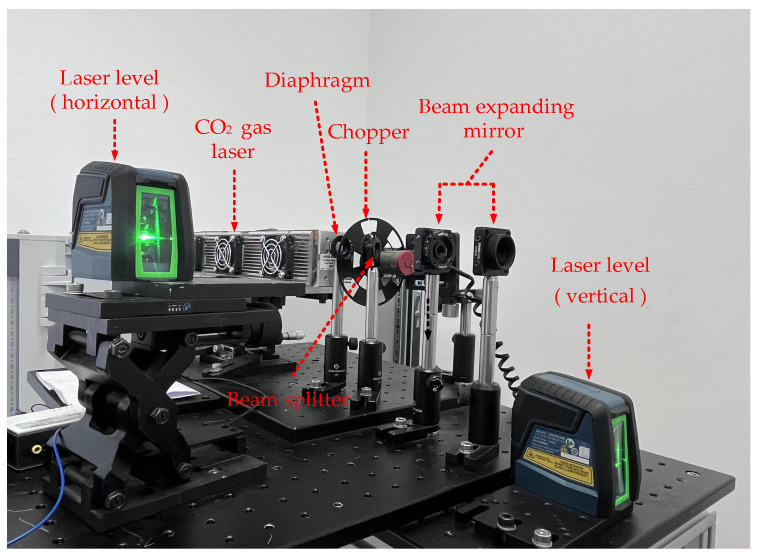
Photo of laser source module.

The laser source module, including laser, collimation and beam splitter–expander paths, is shown in Figure 2. The light source module employs a 10.6 μm CO_2_ laser as the light source, and the optical path is configured with a diaphragm, a chopper, a beam splitter, and a beam expander for adjustment. The diaphragm is employed to regulate the intensity of the beam, with choppers modulating the laser into a fixed frequency of 200 Hz through the filter to remove noise. Beam splitters are used to set up the detection of the optical path for real-time monitoring of the power in situ. Beam-expanding mirrors play a role in making the laser collimation and convergence effect better.

The 10.6 μm mid-infrared laser light was not visible, and it was difficult to regulate the optical path, so the light source module was set up with two laser levels and an infrared color card, which were used to facilitate the observation of the laser irradiation position to regulate the direction of the laser light. The robotic arm holds the sample and moves in space; for the specific rotation method, see our previous paper [27]. Adjusting the optical path so that the laser is always at the center of the sample when the sample moves with the robot arm consists of three steps, described below.

#### 2.3.2. Infrared Laser Adjustment Method

**Figure 3:**
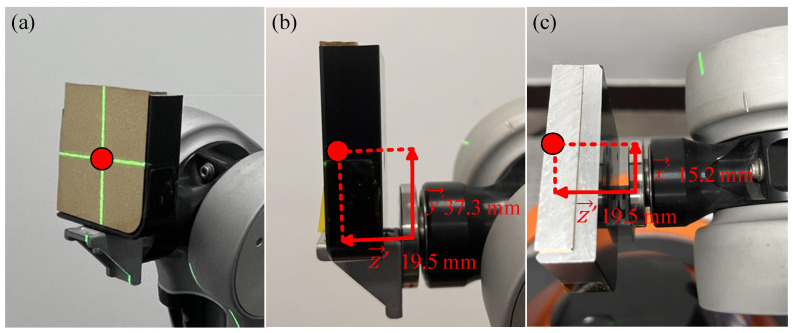
World coordinates of point where sample is illuminated: (**a**) Front view; (**b**) Side view; (**c**) Vertical view.

Step 1. Determine the spatial coordinates of the position point of the laser irradiation on the sample. A world coordinate system ($\vec{x^{\prime }},\vec{y^{\prime }},\vec{z^{\prime }}$) is established at the geometric center of the gripper of the robotic arm to determine the fixed position of the sample to be irradiated, i.e., to determine the position of the sample’s irradiated point in the world coordinate system, as shown in Figure 3. The geometric center of the sample (red dot) is placed at the intersection of the crosses of the level, and the distances between the geometric center of the sample and the geometric center of the gripper of the robotic arm in the $\vec{x^{\prime }}$-axis, $\vec{y^{\prime }}$-axis, and $\vec{z^{\prime }}$-axis directions are measured respectively, so as to obtain the positional coordinates of the center of the movement of the robotic arm gripping the sample in the world coordinate system.

**Figure 4:**
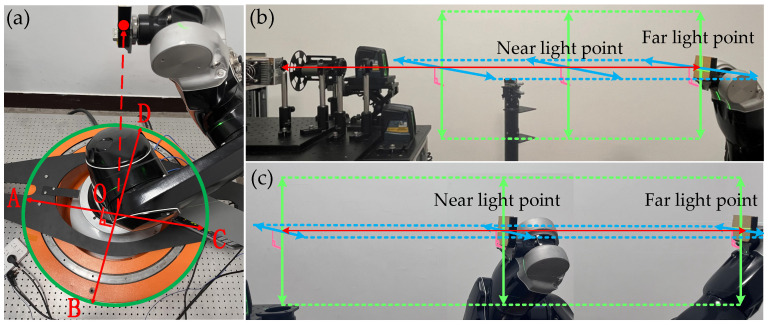
(**a**) Photo of sample vertical projection onto the center of a circular guideway; (**b**,**c**) light path adjustment process.

Step 2. The world coordinate system should be translated so that the sample is centered on the circular guideway. By operating the mechanical arm to translate the world coordinate system as a whole, so that the vertical projection of the irradiated point of the sample is located at point O, which coincides with the center of the circular guideway (OA = OB = OC = OD), as illustrated in Figure 4a, the spatial stereoscopic angle is maintained throughout the rotation of the detector.

Step 3. Adjust the arm pivot axis so that the arm movement axis coincides with the world coordinate system. The intersection line between the vertical plane lasers and the horizontal plane lasers of the two levels is used as the standard direction, which is shown by the red arrow line in Figure 4b. Observe the laser irradiation point at the near light point and far light point through the color rendering card so that the incident light coincides with the intersection line. Adjust the direction of the $\vec{z^{\prime }}$ axis of the world coordinate system to coincide with the direction of the intersection line between the vertical plane laser and the horizontal plane laser of the level meter. As shown in Figure 4c, move the irradiated point of the robotic arm holding the sample along the axis direction of the world coordinate system.

**Figure 5:**
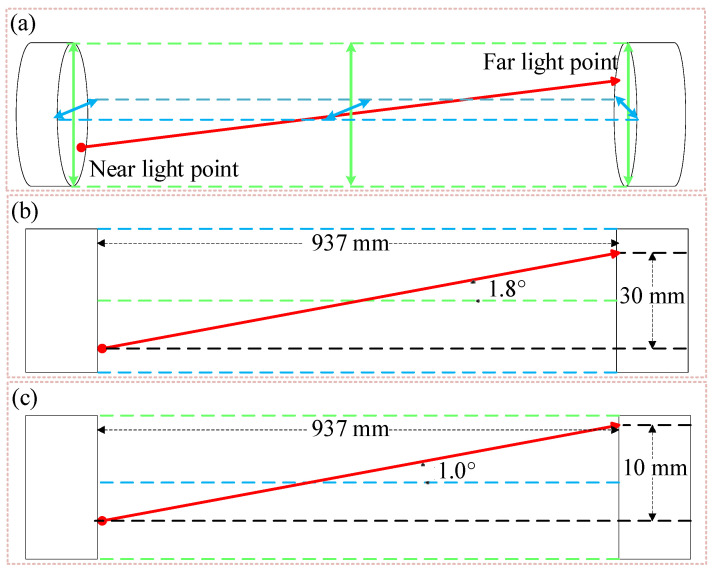
Adjustment process of the $\vec{z^{\prime }}$ axis in the world coordinate system: (**a**) Stereogram; (**b**) Vertical view; (**c**) Front view.

Similarly, use the color card to observe the infrared laser irradiation in the sample near the light point and the position of the far light point, the world coordinate system of the $\vec{z^{\prime }}$ axis pitch, and left–right angle adjustment. The adjustment process is shown in Figure 5a. The adjustment of the pitch and horizontal angles are shown in Figure 5b and Figure 5c, respectively. At this time, the incident light coincides with the z-axis of the world coordinate system, completing the adjustment of the optical path and realizing that the laser is always irradiated at the center of the sample when the robotic arm clamps the sample in motion.

#### 2.3.3. Beam Expansion and Verification

**Figure 6:**
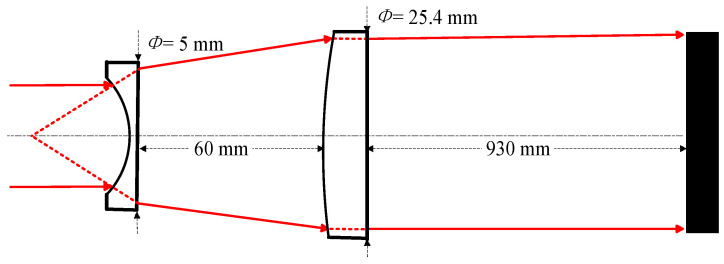
Schematic of beam-expanding optical path.

**Figure 7:**
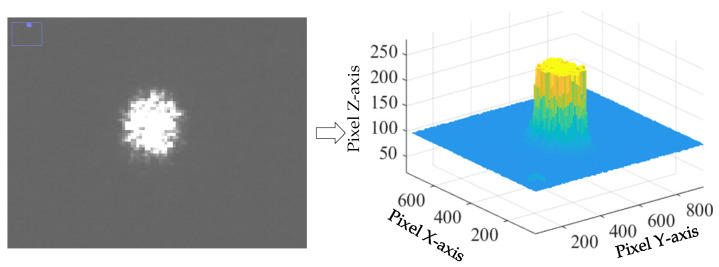
Infrared imaging with beam widening and spot illumination.

The laser beam-expanding optical path uses a combination of a *Φ* = 5 mm diameter plano-concave lens and a *Φ* = 25.4 mm diameter plano-convex lens beam expander, which can transmit wavelengths in the range of 7~14 μm, with a transmittance rate of about 70%, and a beam-expanding factor of about 3~4 times. Parallel infrared laser is first incident to the 5 mm diameter flat concave lens, followed by parallel light beam expansion to the flat convex lens, and then parallel irradiation on the sample, as shown in Figure 6. Using an infrared imager to image the surface of the sample, as shown in Figure 7, the spot diameter after beam expansion is calculated to be about 16 mm, which is in line with the theoretical value, and verifies the effectiveness of beam expansion.

### 2.4. Motion Module

#### 2.4.1. Motion Devices

The motion module consists of a six-axis robotic arm with a circular guideway. The robot arm base is mounted at the geometric center inside the ring guide and consists mainly of the robot arm, the CS9 controller, and the SP2 demonstration box. The robot arm is a six-axis tandem arm with six degrees of freedom, which can realize translation and rotation in the three directions of x, y, and z axes in the coordinate system. The SP2 demonstration Box is used to operate the real-time spatial motion of the robot arm. The SP2 Teaching Box works in the automatic mode and realizes the spatial motion of the robot arm by combining the CS9 controller with the VAL3 simulation software(SRS 2019.2.0) to write the planned motion route of the robot arm through the VAL3 programming language. The circular guideway is mainly composed of servo drive and circular rail. The PAMC control software(PEWIN32 PRO2) of the circular guideway sends rotation commands to the servo drive through the serial port, and the servo drive controls the circular guideway to rotate.

#### 2.4.2. Integrated Controls

**Figure 8:**
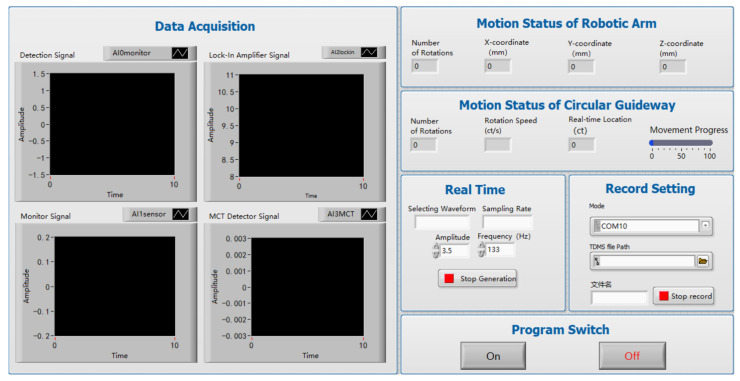
Interface of the controlling program.

Based on the LabVIEW program(NI LabVIEW 2022 Q3) in tandem with the robotic arm VAL3 simulation software and ring track PAMC control software, the control interface is shown in Figure 8. The start of the program switch to complete the prototype is measured on the surface of the hemispherical space of the BRDF of the sample by the automatic measurement of a key.

The program enables the real-time monitoring of the robot arm and circular guideway movements, including the number of robot arm movements, the current coordinates of the tool in world coordinates (x, y, z), the number of rotations of the circular guideway, the current rotation speed of the circular guideway, the real-time position of the circular guideway, and the progress of the circular guideway in completing the movement. In the LabVIEW control interface, data reading, display, and storage functions are also configured. These include the reading function, which displays the current waveform of the collected data; the sampling rate, frequency, and amplitude of the real-time display; and the display function, which shows the real-time changes in the collected data across four channels. The storage function stores the collected data in real time. The application of visualization technology enriches the content of the article and enhances comprehension.

### 2.5. Signal Detection and Processing Module

The signal acquisition module is mainly composed of a detector and a data acquisition card. The detector detects the incident or scattered light in real time and transmits it to the acquisition card, and the data on the acquisition card is transmitted to the host computer through a USB interface. The data are solved in real time by the demodulation program in the host computer, which directly obtains the real-time detected BRDF distribution.

#### 2.5.1. Stray Light Suppression Design

**Figure 9:**
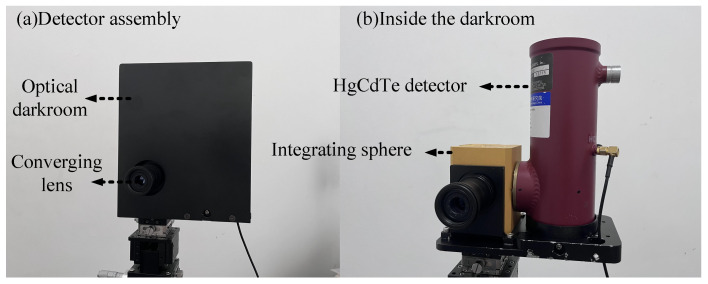
(**a**) Detector assembly; (**b**) interior of the darkroom.

This module uses a HgCdTe detector for incident or scattered light, which is filled with liquid nitrogen and operates at low temperatures for high sensitivity at a wavelength response in the range of 2 to 13 μm. In order to avoid the influence of other stray light during the measurement process, a converging lens, an integrating sphere (diameter of integrating sphere *Φ* = 60 mm, diameter of inlet *Φ* = 20 mm, diameter of outlet *Φ* = 15 mm) and an optical darkroom were designed for the HgCdTe detector. The assembled detector is shown in Figure 9a, with only the converging lens exposed on the outside of the optical darkroom to avoid stray light entering the integrating sphere inlet or outlet junction. Inside the darkroom, as shown in Figure 9b, the converging lens is mounted at the entrance of the integrating sphere, and the exit of the integrating sphere is connected to the detection hole of the HgCdTe detector.

**Figure 10:**
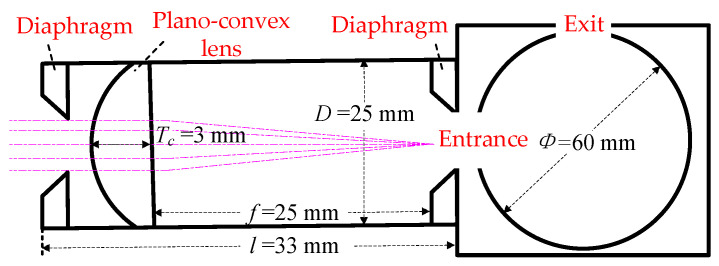
Diagram of light path for converging lenses.

The optical path diagram of the converging lens is shown in Figure 10. The converging lens consists of two adjustable diaphragms (aperture adjustment range 1~12 mm), a plano-convex lens (through the band 8~12 μm, center thickness *T_c_* = 3 mm, focal length *f* = 25 mm), and an optical sleeve (diameter *D* = 25 mm, length *l* = 33 mm). The scattered light from the sample enters into the optical sleeve, and the first diaphragm restricts the incidence diameter of the scattered light. The distance from the plano-convex lens to the entrance of the integrating sphere is equal to its focal length, *f* = 25 mm, i.e., the plano-convex lens converges the scattered light to the entrance of the integrating sphere, which avoids the secondary reflection of the scattered light in the optical sleeve. A second diaphragm is set in front of the integrating sphere entrance to prevent the stray light reflected twice from the optical sleeve from entering the integrating sphere. The scattered light entering the integrating sphere is homogenized, and the detector detects the scattered light at the exit of the integrating sphere.

#### 2.5.2. Correction for Detector Nonlinear Effects

**Figure 11:**
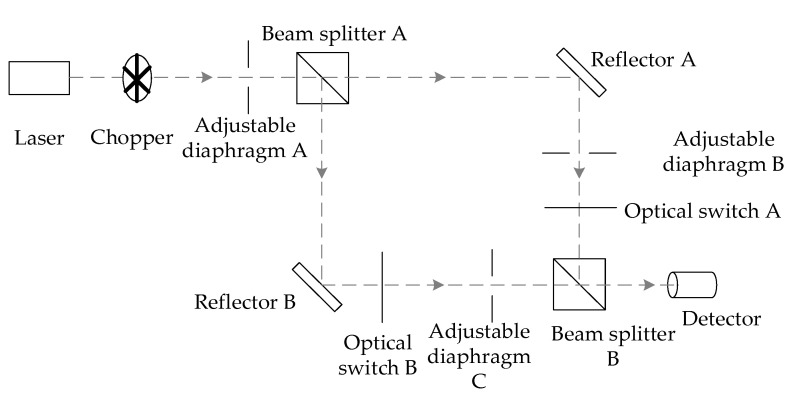
Non-linear effect of the detector. Dichotomous correction of the optical path.

For BRDF measurements of diffuse reflective plates, the signal amplitude of the scattered light is typically several orders of magnitude smaller than the incident light signal amplitude. There is a significant nonlinear effect because the signals are detected using the same HgCdTe detector. In this paper, the optical path is designed based on the optical flux multiplication method and the detector is analyzed for nonlinearity [47,48]. The detector’s nonlinear dichroic correction optical path shown in Figure 11 is constructed to correct the measurement error caused by the nonlinear effect.

After passing through the chopper, the light from the laser is modulated by adjustable diaphragm A to limit the beam diameter. The beam is then split into two equal proportions by beam splitter A. The beam transmitted through beam splitter A is detected by the detector through reflector A, adjustable diaphragm B, optical switch A, and beam splitter B. The light beam reflected from beam splitter A is detected by the detector via reflector B, light switch B, adjustable diaphragm C, and beam splitter B. The light beam is transmitted through beam splitter A by two equal proportional beams. The single-beam and combined-beam measurements of the two beams are controlled by the optical switches A and B. The signal amplitude of the beam via reflector A is noted as *V_A_*, the signal amplitude of the beam via reflector B is noted as *V_B_*, and the signal amplitude of the two beams is noted as *V_A + B_*. The nonlinear coefficient *NL* of the detector is defined as:

(4) $$NL=\frac{V_{A+B}}{V_{A}+V_{B}}$$

where *V_A_*, *V_B_*, and *V_A + B_* are the real output signal amplitude of the detector detecting single-beam or combined-beam light. When the nonlinear coefficient *NL* = 1, the detector output signal amplitude is linear, and when the nonlinear coefficient *NL* ≠ 1, the detector output signal amplitude is nonlinear. When the nonlinear coefficient *NL* < 1, the sensitivity of the detector output signal amplitude decreases with the amplitude order of magnitude; when the nonlinear coefficient *NL* > 1, the sensitivity of the detector output signal amplitude increases with the amplitude order of magnitude.

Changing the aperture of the adjustable diaphragm A controls the intensity of the incident light source, and changing the adjustable diaphragms B and C makes the amplitude of the detected signals of the two single-beam optical circuits equal, even if *V_A_* = *V_B_* = *V*, at which point Equation (4) can be rewritten as:

(5) $$NL=\frac{V_{A+B}}{2V}$$

**Figure 12:**
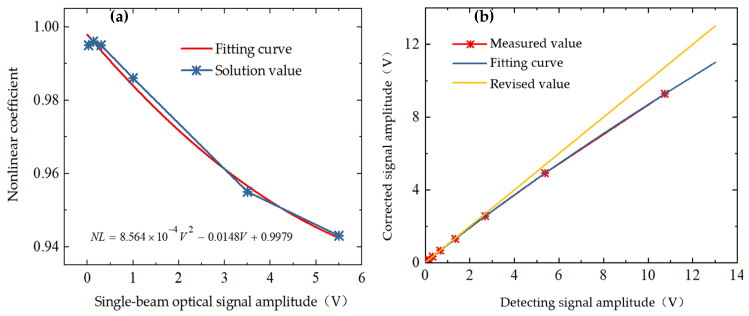
(**a**) Fitting curve of the non-linear coefficients of the detector; (**b**) detector non-linearity correction curve.

Modification of the aperture of the adjustable diaphragm A results in a control of the output range of the single-beam optical path signal amplitude *V* within the range of 0.025 to 5.5 V. Calculation of the nonlinear coefficient of the detector is performed in accordance with Equation (5), as illustrated in Figure 12a. When the single-beam optical signal amplitude *V* is less than 1 V, the nonlinear coefficient is close to 1, and the detector output signal amplitude is close to linear; when the single-beam optical signal amplitude *V* is greater than 1 V, the nonlinear coefficient gradually decreases, and the detector output signal amplitude sensitivity increases and decreases by orders of magnitude. When the single-beam optical path signal amplitude *V* = 5.5 V, the nonlinear coefficient decreases to 0.943. A quadratic polynomial is fitted to the nonlinear coefficient to obtain an approximate nonlinear coefficient curve as:

(6) $$NL=8.564\times 10^{-4}V^{2}-0.0148V+0.9979$$

Combining Equations (5) and (6), a cubic polynomial was fitted to the nonlinear coefficients by NL for specific values during the measurement process, and the cubic fitted curve expression is:

(7) $$NL=-1.988\times 10^{-4}V^{3}+0.0179V^{2}+1.01V-4.978\times 10^{-4}$$

The correction signal amplitude is obtained by correcting the true output detection amplitude of the detector through Equation (7), and the correction curve is shown in Figure 12b.

### 2.6. Temperature Controlling Module

#### 2.6.1. Development of a Lightweight Heater

**Figure 13:**
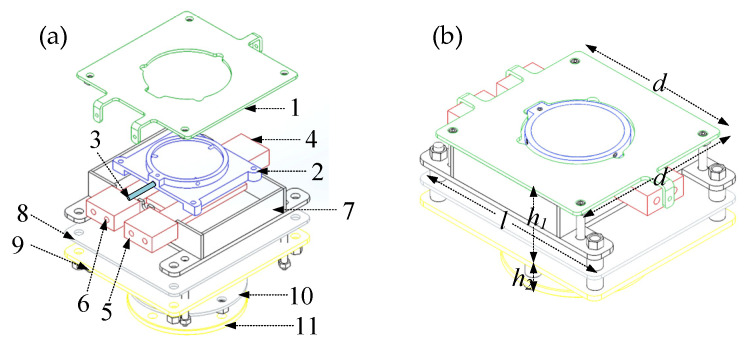
(**a**) Exploded view of the heater. 1 Heater top cover, 2 Heating tank, 3 Thermocouple, 4–6 heating plate, 7 Insulation shell, 8–11 Insulation layer. (**b**) Physical view of the heater.

The structure of the heater used in this paper is shown in Figure 13a. The core part of the heater is the heating groove. In the upper part of the heating groove, a circular groove with a diameter of 50 mm, depth of 5 mm, and wall thickness of 3 mm is cut out. A sample with a diameter of 50 mm and thickness of 5 mm is embedded inside the circular groove, and the side wall of the circular groove is tapped with three equidistant threaded holes with a diameter of 3 mm, which are used for spatial limitation by pressing the sample through the screws. The spatial limitation is achieved by screwing the sample piece.

**Figure 14:**
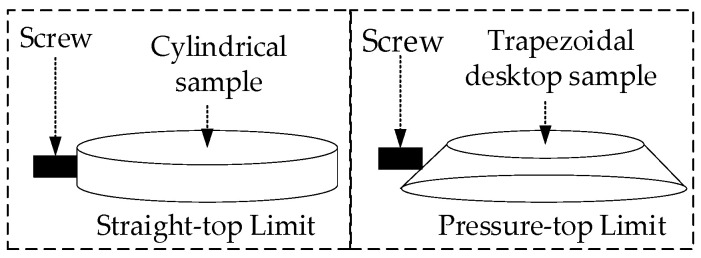
Schematic of measured sample.

As a result of thermal expansion and contraction effect, the heating and cooling of the sample causes the screws to produce a gap; as a result, the sample will be processed into a trapezoidal desktop sample, i.e., the three screws of the straight top effectively prevent the heated and cooled sample from falling off. A schematic diagram of the sample limit is shown in Figure 14. Similarly, a through-hole with a diameter of 3 mm is tapped into the side wall of the circular groove, and an S-type thermocouple with a diameter of 2.5 mm is inserted into it to contact the sample and measure the temperature of the sample in real time; the temperature is then fed back to the LabVIEW-PID controller for real-time temperature control. The lower part of the heating slot is cut with a rectangular groove of 60 mm in length, 50.3 mm in width, 5 mm in depth, and 3 mm in wall thickness. Three parallel silicon carbide heating rods, each with a power of 400 W, are inserted side by side into the rectangular groove. Since the maximum temperature of the sample heating is 1000 °C, the material of the heating tank is stainless steel GH2747, which can withstand temperature up to 1250 °C. The assembled heating bath is fixed in the heater shell, and the empty space in the heater shell is filled with asbestos for heat preservation. A 55 mm diameter circular hole is cut into the top cover of the heater, which is installed to limit the space of the heating rod and keep its movement stable. After assembling the heating tank, sample, thermocouple, heating plate, heater top cover, and shell, the whole unit is mounted on the four-layer heat insulation board. The heater assembly is shown in Figure 13b, where the heater assembly has length *l* = 12.4 cm, width *d* = 10.0 cm, height *h*_1_ = 4.80 cm and height *h*_2_ = 2.60 cm.

#### 2.6.2. Design of PID Temperature Control System

The temperature of the sample is read by the S-type thermocouple and transmitted to the PID controller for real-time temperature control. The PID controller consists of a DC power supply, a digital multimeter, and LabVIEW-PID control software (NI LabVIEW 2022 Q3). According to the potential difference as a function of temperature, the dividing table of the thermocouple is obtained, and the equation of temperature as a function of potential difference is calculated through the dividing table as:

(8) $$T=30.0199U^{3}+122.8413U^{2}-3.0452U+0.009$$

where *T* (°C) is the temperature, and *U* (mV) is the potential difference. An S-type thermocouple is used to read the temperature of the heater. The working end and the free end of the potential difference is measured with a KEITHLEY-2000 digital multimeter. The value of the potential difference through the serial port is fed to the LabVIEW-PID control software within the real-time temperature of the sample calculated according to Equation (8).

The PID parameters in LabVIEW-PID control software are calculated by the critical proportion method, where *K_p_* denotes the proportional coefficient, which reacts to the current deviation of the system from the set value and can regulate the speed of offsetting the error. *T_i_* denotes the integral coefficient, which reacts to the accumulated deviation of the system, and can offset the error in real time until it disappears. *T_d_* denotes the differential coefficient, which reacts to the rate of change of the deviation of the system. The trend in the deviation can be predicted. In the PID critical proportion method, the integral coefficient *T_i_* is initially set to infinity, the differential coefficient *T_d_* is set to 0, and the proportionality coefficient *K_p_* is gradually increased from a low value to a high value. The temperature curve is then observed to determine whether the oscillation is attenuated. If the oscillation is attenuated, the proportionality coefficient *K_p_* is increased until the oscillation is dispersed. If the oscillation is dispersed, the proportionality coefficient *K_p_* should be reduced until the temperature curve appears to have an equal amplitude of oscillation. At this juncture, the proportionality coefficient *K_pr_* should be recorded, as well as the temperature curve and the oscillation period *T_r_*. According to the empirical formula, *K_p_* is set to 0.6*K_pr_*, *T_i_* is set to 0.5*T_r_*, and *T_d_* is set to 0.125*T_r_*.

**Figure 15:**
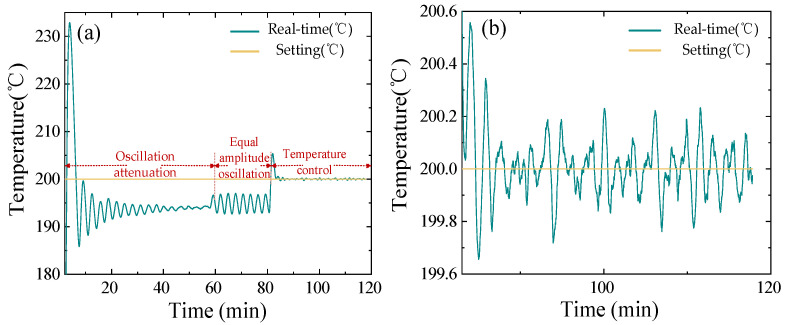
(**a**) Temperature profile during commissioning stage of PID parameters. (**b**) Temperature curve of the temperature control stage.

Taking the set temperature of 200 °C as an example, as shown in Figure 15a, the temperature curve oscillates and decays during the time period of 0~60 min and increases to *K_p_* = 5.5. The temperature curve oscillates with equal amplitude during the time period of 60~80 min, and is recorded at *K_pr_* = 5.5 and *T_r_* = 2.83 min, respectively. Then, according to the empirical formula, the PID parameters are set to *K_p_* = 0.6 *K_pr_* = 3.3, *T_i_* = 0.5 *T_r_* = 1.415 min, *T_d_* = 0.125 *T_r_* = 0.354 min, respectively. After setting the PID parameters, the temperature rises and stabilizes at 200 °C. The temperature control process is illustrated in Figure 15b, and the maximum temperature deviation from the set temperature is 0.55 °C, which represents a favorable outcome in terms of temperature control.

## 3. Experiments

### 3.1. Room Temperature BRDF Measurement 

To ensure the accuracy of the BRDF measurements, the experiments in this study were conducted in a controlled laboratory environment where environmental factors were ignored and not used as experimental variables [49,50].

**Figure 16:**
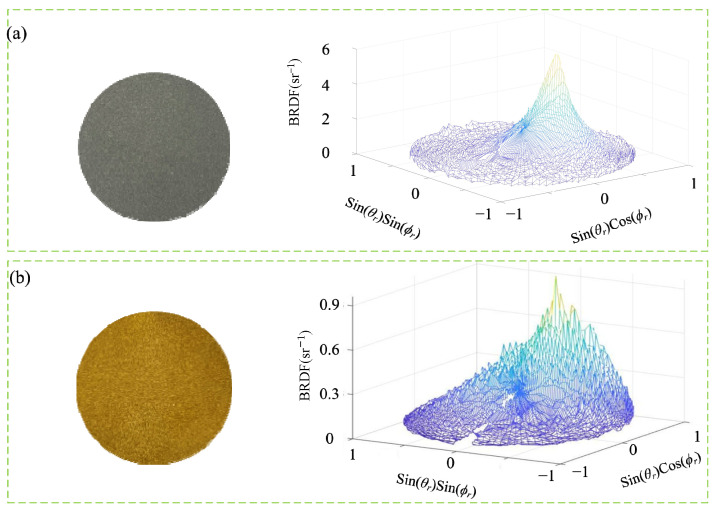
(**a**) Plated sample and its BRDF distribution. (**b**) Gold plate and its BRDF distribution.

In this section, the validity of the system is verified by measuring the BRDF of specular and diffuse reflection samples at room temperature. The samples measured at room temperature are plated samples and gold plates, and the plated samples are typical specular reflection samples. Under the condition of incident zenith angle *θ_i_* = 30°, the physical map of the samples and the three-dimensional BRDF distributions are shown in Figure 16a. It can be observed that the scattered light in the hemispherical space of the sample has obvious strength; the peak position of the BRDF is the stronger position of the scattered light, and the lowest position of the BRDF is the weaker position of the scattered light. Under the same incidence conditions, the gold plate is a typical diffuse reflection sample; its sample physical map and three-dimensional BRDF distribution shown in Figure 16b. The sample’s hemispherical space scattered light is still strong and weak respectively, but compared to the plated sample, its scattered light in the sample hemispherical space has a certain component, whereby the three-dimensional BRDF distribution map represents the projection of the BRDF distribution in the hemispherical space of the sample to the two-dimensional plane (x, y) in which the sample is measured, denoted as:

(9) $$\left\{\begin{array}{c} \vec{x}=\sin\theta_{r}\sin\phi_{r}\\ \vec{y}=\sin\theta_{r}\cos\phi_{r} \end{array}\right.$$

### 3.2. Variable Temperature BRDF Measurement 

#### 3.2.1. SUS314 Sample Preparation and Measurement Process

The sample used for variable temperature experiments in this paper is an industrially common high-temperature alloy material stainless-steel SUS314, with high temperature and oxidation resistance, especially suitable for applications in high-temperature environments, such as furnace tubes, heat-treatment equipment, chemical equipment, etc., which has a certain research value [51]. The study of spectral emissivity and BRDF of high temperature alloys is an effective method to better understand their performance characteristics [52]. It is not currently feasible to conduct spectroscopic thermal emissivity measurements due to the constraints of the laboratory conditions, but these will be carried out in future studies.

**Figure 17:**
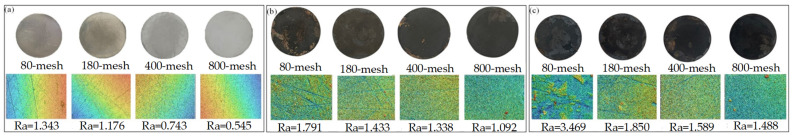
Appearance and microstructure of samples with different roughness: (**a**) unheated; (**b**) after initial heating; (**c**) after secondary heating.

Four stainless-steel 314 samples (SUS314) with a diameter of 50 mm and a thickness of 5 mm, which can withstand temperatures up to 1050 °C, were measured in experiments. Four samples were polished using a metallographic grinding machine. The sandpaper used for polishing were 80 mesh, 180 mesh, 400 mesh, and 800 mesh, and the polished samples are shown in Figure 17a. With the increase in the grit of the sandpaper, it can be directly observed that the scratches are less obvious. The microstructure of the four samples was observed and the surface roughness was measured using a 3D morphometer, and the microstructure of each sample is shown in Figure 17a. In the microstructure of the same area, the surface roughness (average roughness, Ra) of each sample decreases gradually with the increase in the grit of the sandpaper: Ra = 1.343 μm, Ra = 1.176 μm, Ra = 0.743 μm, Ra = 0.545 μm, respectively; the surface microstructure of the 80-mesh sample can be observed in the width and depth of the obvious scratches. Compared to the 80-mesh sample, the surface microstructure of the 800-mesh sample shows obvious fine and shallow scratches, i.e., with a decrease in roughness, the less obvious the scratches in the microstructure of the same area become.

**Figure 18:**
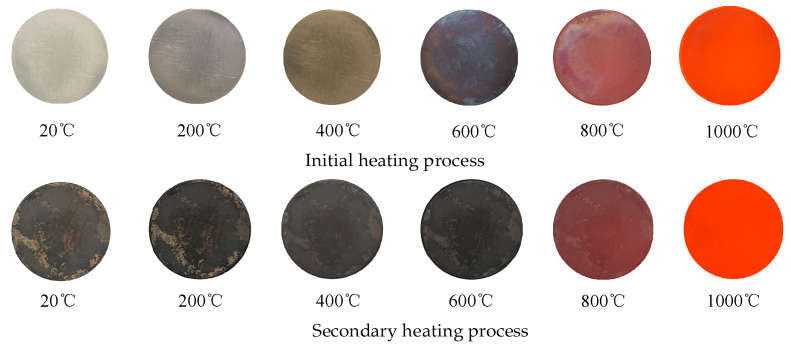
Surface change of 800 mesh samples when heated to 1000 °C for the first and second time.

The measurement process of each sample was divided into primary and secondary heating, and the three-dimensional BRDF distribution in the hemispherical space on the sample surface was measured at 20 °C, 200 °C, 400 °C, 800 °C, and 1000 °C during the primary and secondary heating process to compare the changes in the spatial distribution of the BRDF and to record the changes in the peak value of BRDF in the hemispherical space of the samples with the increase in temperature. The detection method of scattered light in hemispherical space adopts a variable angular interval detection model, which detects the scattered light with a resolution of 0.2° in the region where the peak of BRDF is concentrated, and detects the scattered light with an angular resolution of 10° in the range where the BRDF value is relatively small. Taking the 80-mesh samples as an example, the samples were stabilized at each temperature for more than 30 min, demonstrating changes in appearance during the initial and secondary heating as shown in Figure 18. In the initial heating process, from room temperature (20 °C) heated to 200 °C, the sample surface shows no obvious changes. At 400 °C, when the surface of the sample slowly oxidized, the color is yellowish; at 600 °C, the sample surface forms a blue oxide film; at 800 °C, the area of the blue oxide film decreases and the surface of the sample is slightly bright; and at 1000 °C, due to thermal radiation of the sample, it is red and bright, and the real appearance of the sample surface cannot be observed. After the initial heating to 1000 °C, the sample cooled, which allowed us to observe the surface microstructure, measure the surface roughness, and compare the surface microstructure and surface roughness with the unheated sample to analyze the relationship between the BRDF and temperature change and microstructure (surface roughness). The microstructures of the four samples that were initially heated to 1000 °C and then cooled to room temperature were observed with a 3D morphometer, and the physical drawings and microstructures of the samples are shown in Figure 17b. After the initial heating, compared with the microstructure of the unheated samples, the metallic luster on the surface of each sample disappeared and a dark gray oxide layer was generated. The surface roughness of each sample increased to different degrees, while the surface scratches faded to different degrees.

In the secondary heating process, we again measure the BRDF value at different temperatures and compared the change rule with the unheated and initial heating samples to analyze the change principle. As shown in Figure 18, when the sample is heated to 400 °C, the sample surface shows no obvious changes and is dark gray; at 600 °C, the sample surface becomes dark black; at 800 °C, the sample surface is slightly bright, with part of the oxide layer sloughed off; at 1000 °C, as before, the real appearance of the sample surface cannot be observed. Comparing the physical map and microstructure of the sample surface after cooling after the second heating, as shown in Figure 17c, the dark gray oxide layer on the surface of each sample turns into dark black after the second heating, and part of the oxide layer falls off. The surface roughness of each sample has increased to a certain extent, but the change in surface scratches is not obvious, i.e., the microstructure of the surface has not changed much and is not easy to observe.

#### 3.2.2. Measurement of BRDF During Initial Heating of SUS314 Samples

**Figure 19:**
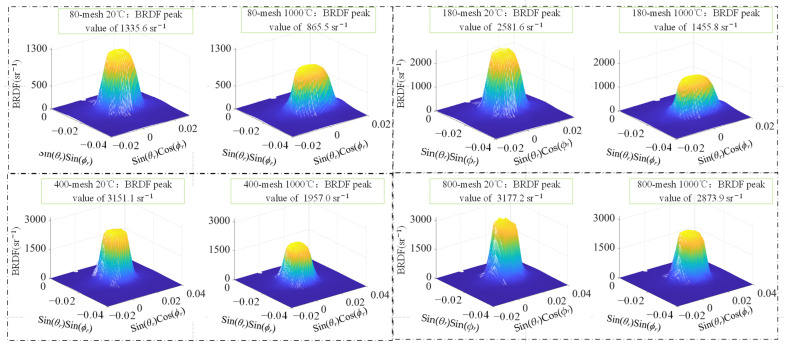
BRDF distributions of samples with various roughness at initial heating to 20 °C and 1000 °C.

During the initial heating process, the spatial BRDF distribution of hemispherical space on the sample surface was measured for four samples at 20 °C, 200 °C, 400 °C, 800 °C, and 1000 °C. The spatial distribution of the peak BRDF region of the four samples with different roughness at 20 °C and 1000 °C is shown in Figure 19. Since the reflection characteristics of the four samples are specular reflection characteristics, the peak region of the spatial distribution of the three-dimensional BRDF changes significantly during the heating process, and the other spatial distribution components do not change significantly, so the three-dimensional BRDF distribution of the four samples only shows the region near the peak. The four samples from 80 mesh to 800 mesh showed 35.2%, 43.6%, 37.9%, and 9.5% reduction in BRDF peaks during the initial heating process from 20 °C to 1000 °C, respectively.

**Figure 20:**
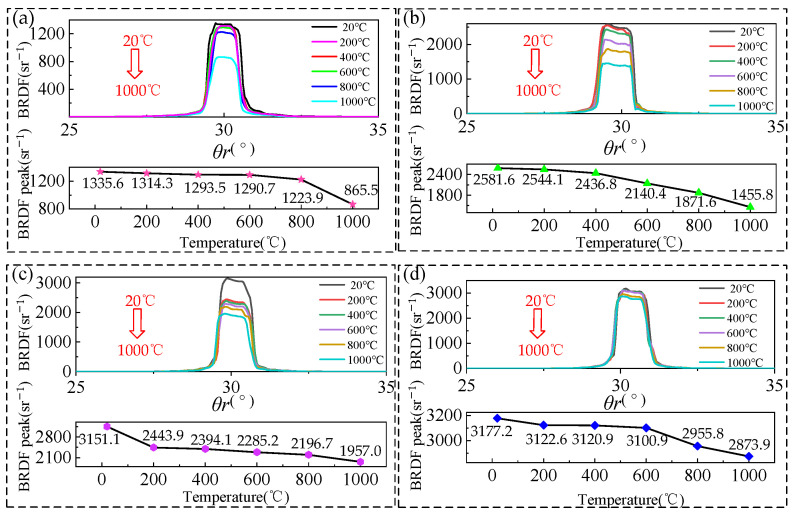
Variation in BRDF peak values with temperature at initial heating of samples with different roughness: (**a**) 80-mesh sample; (**b**) 180-mesh sample; (**c**) 400-mesh sample; (**d**) 800-mesh sample.

To more clearly demonstrate the manner in which the BRDF changes with temperature, the BRDF distribution at different temperatures was extracted in order to obtain the maximum peak BRDF change curve under a fixed reflection azimuth, as shown in Figure 20. During the initial heating process, the BRDF peak value under the fixed azimuth angle of the four samples decreases with the increase in temperature, but the BRDF reduction rate of the samples with different roughness changes differently with the increase in temperature. In the process of heating from 20 °C to 1000 °C, the BRDF peak reduction rate of 80 mesh and 180 mesh samples is initially smooth and then abrupt; for 400 mesh and 800 mesh samples, the BRDF peak reduction rate is initially abrupt and then smooth, or the whole process of change shows a very smooth state.

The impact of temperature on BRDF can be ascribed to alterations in the surface morphology and chemical composition of the sample. The temperature at which oxidation occurs on the surface of samples with different roughness is distinct, as is the degree of change in surface morphology and chemical composition caused by surface oxidation at the same temperature. Consequently, the BRDF reduction rates of these samples vary with increasing temperature. Following the initial heating to 1000 °C and then cooling to room temperature, the four samples were observed through the 3D morphometer. Combined with the rule of change in the BRDF peak, with the increase in temperature, the 80-mesh and 180-mesh samples show a greater degree of destruction of the surface microstructure. As the sample surface roughness increases, so does the increase in the degree of destruction of the surface microstructure, and the BRDF peak rate of reduction shows a more drastic trend. For 400-mesh and 800-mesh samples, any time the temperature increases, the sample surface roughness increases, the degree of destruction of the surface microstructure is not obvious, and the BRDF peak reduction rate presents a more gentle trend.

#### 3.2.3. Measurement of BRDF on Secondarily Heated SUS314 Samples

**Figure 21:**
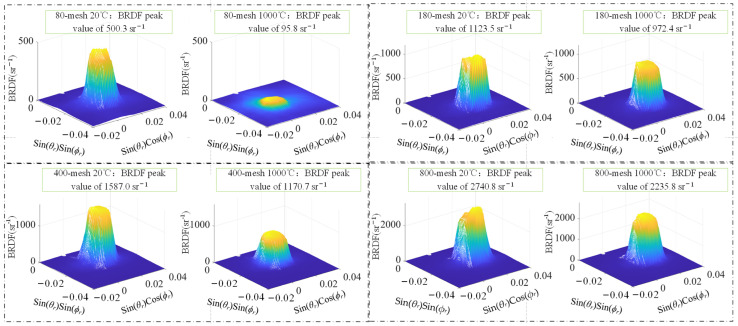
BRDF distributions of samples with various roughnesses at secondarily heated to 20 °C and 1000 °C.

After the initial heating of the samples, the four samples were cooled to 20 °C, and the BRDF spatial distribution of the samples was measured, as shown in Figure 21. Compared with the BRDF spatial distribution when the samples were initially heated up to 1000 °C and then cooled to 20 °C, the peak portion of the BRDF spatial distribution changed from smooth to bumpy. The BRDF peaks of all four samples decreased to different degrees after cooling. It was observed that the surface oxide layer was detached after the sample was cooled, and it was believed that the oxide layer detachment damaged the surface structure of the sample, which made the BRDF peaks continue to decrease during the cooling process. The spatial distribution of the three-dimensional BRDF peak regions of the four samples at 20 °C and 1000 °C during the secondary heating process is shown in Figure 21. The four samples from 80 mesh to 800 mesh showed 80.9%, 20.5%, 26.2%, and 18.4% reduction in BRDF peaks during the second heating process, respectively. During the second heating process, the surface roughness of each sample increased, in line with the law that the BRDF value of the sample surface decreases with the increase in roughness.

**Figure 22:**
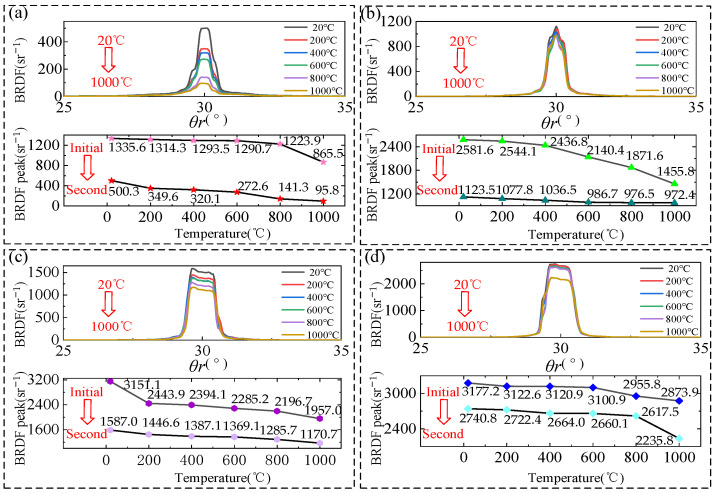
BRDF peak versus temperature during second heating of samples with different roughness: (**a**) 80-mesh sample; (**b**) 180-mesh sample; (**c**) 400-mesh sample; (**d**) 800-mesh sample.

Similarly, to elucidate the alteration in BRDF with temperature during the second heating process of the sample, the change curves of the peak value of BRDF at varying temperatures are plotted, and the transformation of the peak value of BRDF throughout the two heating processes is compared, as illustrated in Figure 22. The average reduction rate of BRDF peaks of 80-mesh samples and 180-mesh samples during the second heating process is more gentle compared with that during the initial heating process, and there is no longer a drastic reduction in BRDF peaks. Comparing the surface microstructure of the samples cooled after the second heating, as shown in Figure 17c, with the surface microstructure after the initial heating and cooling, the scratches are not obvious, i.e., there is not much change in the surface microstructure, which is in line with the situation that the reduction rate in the peak BRDF of the samples is very gentle during the second heating process. In 400-mesh and 800-mesh samples heated from 20 °C to 1000 °C, the whole process of BRDF peak reduction rates is maintained at a flat rate. In the same comparison of the surface microstructure of the samples cooled after the second heating, and the initial heating and cooling of the surface microstructure of the samples after the initial cooling, the change is not much, in line with the samples in the initial and second heating process of the BRDF peak in the reduction rate, which shows a relatively flat rate.

In summary, during the initial heating process, with the increase in temperature, the surface structure of the samples is damaged. Higher temperature causes greater damage to the surface structure, and the BRDF values of the samples show a sharp decrease with the increase in temperature. For the samples with large surface roughness, the high temperature destroys the surface structure more violently, then the BRDF value of the samples with large surface roughness decreases violently with the increase of temperature, and the surface roughness of the small samples of the BRDF value of the temperature with the increase of the slow decrease. After heating samples cooled to room temperature back up to 1000 °C, the shedding of the sample’s surface oxide layer led to the continued destruction of the surface structure. During the process of initial heating and cooling to room temperature, the BRDF peak continues to decrease. During the second heating process, high temperature does not cause obvious damage to the surface structure of samples with different roughness; with the increase in temperature, the BRDF value shows a gentle decrease.

## 4. Discussion and Conclusions

In this paper, a variable-temperature infrared BRDF measurement system was developed for high-resolution measurement of BRDF in the mid-infrared band under variable-temperature conditions. Aiming at the problem of invisible mid-infrared light, the collimation of the light source is adjusted by a laser level and an infrared color-rendering card, a beam-expanding light path is constructed, and the light spot irradiated on the surface of the sample is imaged by the infrared imager to verify the validity of the beam-expanding light path. LabVIEW software facilitates the integration of the robotic arm and ring rail control programs, enabling unified control of both systems. Additionally, the signal acquisition, storage, and processing are concurrently coupled into LabVIEW, allowing for high-resolution measurements of the hemispherical BRDF on the measured surface of the sample piece. In the signal processing process, the optical convergence lens and optical darkroom are designed for the HgCdTe detector to effectively suppress the influence of stray light, and linear calibration of the detector is carried out to offset the nonlinear error of the detector’s response coefficients to different light sources of different intensities. In the process of variable temperature measurement, for the heating and temperature control of the sample, a portable heater suitable for the mechanical arm corner mechanism is used, together with a DC power and a digital multimeter, to determine the temperature control parameters through the critical parameter calibration method, to realize that the maximum error of temperature control is no more than ±1 °C.

The BRDF distributions of typical specular reflection samples and diffuse reflection samples were measured at room temperature to verify the validity of the measurement system. The BRDF distributions and peak versus temperature curves of four SUS314 stainless-steel samples with different roughness were measured at variable temperatures with two heating experiments at six temperature nodes under the condition of incident zenith angle *θ_i_* = 30°. Concurrently, the trends in the physical and microstructural maps of the unheated, primary and secondary heated samples were observed and analyzed, and the mechanism of the BRDF changes in samples of different roughness in different heating processes is summarized. During the initial heating process, the high temperature destroys the surface structure of the sample with large roughness more suddenly, and its BRDF value decreases suddenly with the increase in temperature. In the sample with small surface roughness, the BRDF value of decreases gently with the increase in temperature. In the second heating process, the high temperature is not obvious to the destruction of the surface structure of the samples, and the BRDF value of the four samples decreases gently with the increase in temperature.

The variable temperature infrared BRDF measurement system was developed for the purpose of evaluating the optical properties of high-temperature materials and coating materials within the field of material science [53,54]. Additionally, the system is intended to optimize the surface design of solar collectors in energy technology [55] and to provide a research basis for improving the accuracy of remote sensing in remote sensing technology [9,12]. However, the system still has limitations and potential challenges. For example, the measured wavelength is limited by the laser light source, which can only obtain the BRDF distribution under a 10.6 μm light source; the light source can be replaced with different wavelengths in subsequent studies. In addition, the current system can measure data at a maximum of 1000 °C, and further design of heaters with a larger temperature range and suitable for robotic arms can be considered in the future.

## Figures and Tables

**Table 1:** Summary of bidirectional reflectance distribution function (BRDF) measurement devices and parameters at ambient temperature.

Motion Type	Wavelength	Reflected Azimuth Range	Reflected Zenith Angle Range	Spatial Angular Resolution	Ref.
Unmanned Aerial Vehicle	350~2500 nm	0°~330°	0°~36°	6°	[16,17,18,19]
Closed-field	380~780 nm	0°~360°	0°~80°	1°	[21,23]
Multi-rotor	400~2400 nm	0°~360°	0°~90°	0.09°	[4,24]
Six-axis robotic arm	500~2450 nm	0°~360°	0°~90°	0.02°	[34,35]

**Table 2:** Summary of variable temperature BRDF measuring devices and parameters.

Heating Method	Maximum Heating Temperature	Applicable Cornering Mechanism Type	Ref.
Nd:YAG solid state laser	850 °C	Closed field	[36,37]
CO_2_ gas laser	500 °C	Closed field	[38,39]
Cylindrical heating furnace	500 °C	Multi-axis	[40,41]
Flat plate heaters	800 °C	Multi-axis	[42,43]

**Table 3:** Main equipment and parameters of the proposed BRDF measurement system.

Equipment	Model Number	Parameters
Portable heaters	Custom made	Maximum heating temperature 1000 °C; Temperature control error ± 0.7 °C
CO_2_ gas laser	Access Laser-L4S	Maximum power 1.5 W; Wavelength 10.6 μm
Chopper	MODEL SR-540	Operating frequency 40~4000 Hz
Six-axis robotic arm	STAUBLI-TX2-60	Load 3.5 kg; Operating temperature 5~40 °C.
Circular guideway	MicroFlex e190	Load 20 kg; Operating temperature 5~40 °C.
Integrating sphere	Custom made	Outside diameter 6 cm; entrance diameter 2 cm
HgCdTe detector	InfraRed Associates-MCT- 13–4.00	Fixed stereoscopic corners 0.0013 sr Wavelength response range 2–13 μm
Data acquisition card	NI USB-4431	Sampling frequency 102.4 kHz

## Data Availability

Data will be made available on request.
